# A Case in Practice

**Published:** 1891-02

**Authors:** J. G. W. Werner


### A CASE IN PRACTICE.[1]

BY J. G. W. WERNER, D.M.D.

Mr. President and Gentlemen,—There is always an unusual interest in those cases that do not occur every day,—that come to us only occasionally and have in them the possibilities of seriousness or even danger. The case that I am about to relate is one of that nature; one that was of considerable pain, trouble, and anxiety to the patient, and greatly interested the operator.

Mrs. D., sixty-seven years old, came to me July 23, 1888, suffering from pain in the right superior maxillary region. Some three years previously I had extracted a loose root from her right upper jaw. It seemed to be the remnant of the third molar. I remember well this extraction, for with it came almost all of the alveolar socket, which was in a soft, black condition, the root itself having exostosis. The wound from this extraction was much longer in healing than in an ordinary case.

Before the patient came to me, as above mentioned, there had been for some time previously a feeling of heaviness, of congestion, of more or less dull, grumbling pain, as if something was wrong deep in the jaw.

On waking one morning, the patient found her pillow stained with saliva and pus, and had a decidedly bad taste. Something had wakened her,—a noise in or near the ear, as if something had broken, followed by sharp twinges of pain. It was after this that she consulted me. On the right side of her face, near the mouth and chin, the skin had a peculiar reddish eruption and was in a high state of irritation. As the patient said, "I was frightened to find my face all broken out, never having had anything like it."

After a few days this irritation and eruption subsided. From the time the patient felt this something giving way, the sharp snap and those twinges of pain, there was considerable pus discharged into the mouth and throat.

As stated, I saw the case first on July 23, and, from the gathering of symptoms, I thought at once either of abscess involving the submaxillary sinus, the inner ear, or the post-nasal and palatal regions.

On examination, the right nostril and the right ear seemed to

---

[1] Read before the Harvard Odontological Society, December 31, 1890.

be in normal condition, excepting the peculiar noise the patient heard at times in the ear.

On the right upper jaw there was no tooth posterior to the cuspid, that, with the lateral and central incisors, being the only teeth in position. The roof of the mouth was normal, though very flat. The right tonsil was considerably larger than the left; the soft parts between the opening of the parotid-gland duct and the right tonsil were somewhat red and congested. From whence, then, came the pus, the secretion? Apparently there was no opening, and a searching examination, lasting for nearly an hour, did not reveal the cause or the seat of the trouble. Was it abscess, or chronic ulceration of the tonsil, or a suppuration of the parotid gland? Did the pus come through Steno's duct? All this was considered and the parts thoroughly examined. I had nearly given up searching, when, to my great surprise, I found a small opening, situated at the very end of the maxillary bone, at its junction with the soft palate. Into this opening I could press, by using considerable force, the end of a small round burnisher. This much gained at the first sitting, the patient was dismissed with the following:

℞ Acidi carbolici, ʒ iii;

Listerine, ℥ iv;

Glycerini, ℥ ii. M.

Sig.—One teaspoonful in a glass of warm water as a mouth-wash.

On the following day, July 24, the eruption on the face was at its worst. The feeling of a general fulness and uneasiness about the jaw, the ear, and the throat, was as on the preceding day, but there had been no sharp pain, no special noise or snap as if something had given way, or, in other words, there had been considerably less pus discharged in the past twenty-four hours.

I was anxious at the second sitting to arrive at a more definite and satisfactory understanding of the case. It was either a case of abscess in the antrum, a supernumerary and undeveloped tooth, or necrosis of the maxilla, pointing, in my opinion, strongly towards the latter. With suitable probes, I found I could pass in two directions, directly posterior to an extent of over an inch without perceptible obstruction, and about the same distance laterally towards the median line, in the direction of the suture of the two maxillæ, but with some obstruction.

With a lancet the small opening in the soft tissues was enlarged, the whole cavity washed out with warm water, carbolic acid, and oil of cloves. Into the enlarged opening a large pellet of cotton, saturated with listerine, was crowded. This crowding of cotton into the cavity proved an effectual means of keeping open and further enlarging the orifice, thereby making the examinations easier. The patient was also given instruments and instructions for syringing and packing the cavity every sixth hour, or oftener.

I saw and treated the case daily between the 23d of July and August 1, during which time a considerable number of small pieces of dark fetid bone were loosened and removed. The cavity in the maxilla had now become quite large. With a probe I could now feel something that surprised me, that felt to my touch like the enamel of a tooth. My diagnosis was completed. It was so conclusive to me that then and there I wrote on a piece of paper the following: "Mrs. D. has a supernumerary tooth embedded in the right upper maxillary bone, near the tuberosity, and likely connecting with the antrum. I shall remove it on Friday next." This I gave to the patient with the request to hand it to her family physician and to let me know if he would like to be present and assist me in the operation.

It was the wish of her physician that I should perform the operation, and, being unable to be present or assist me, I procured the assistance of Dr. E. B. Hitchcock. On August 3, 1888, the tooth and pieces of bone you see in this bottle were removed. To extract this tooth from its embedded and inaccessible locality was a very difficult thing to do. It was accomplished after many trials and two hours of hard work. It could have been more easily done if more gum and bone-tissue had been sacrificed, but to avoid doing this was a prime consideration. First, the opening, which was less than half an inch in diameter, was enlarged sufficiently with a bistoury to insert a half-open extracting forceps. Then, with excavators and forceps, small pieces of the bone were removed. In this way the tooth, of which at first only one cusp could be felt, was made more accessible, and eventually became sufficiently loosened to be moved or drawn down considerably. By degrees enough of the bone obstruction was cut away to grasp the tooth with an extracting forceps, with which it was removed. All the roughened surfaces in the maxillary bone were scraped smooth and the whole cavity thoroughly washed out, then packed with cotton saturated with listerine and oil of cloves.

I might mention here that the patient went through the whole operation without an anæsthetic, though everything was prepared for etherization; but the very fact of the patient being conscious assisted very much by enabling us to work more carefully and intelligently. Very few patients would have stood it without ether, for while the greater portion of the operation was not very painful, some of it was.

The day following the operation, August 4, I dressed the wound; also on August 6. Owing to absence from the city, I did not see the case again until August 22.

The condition of the patient, of the local parts, and the after-treatment, may be summarized as follows: After the operation the patient was considerably exhausted, though, considering her age, her condition was very good. On reaching home, nourishment was taken. She then retired and slept part of the night. The following day patient was up part of the time. In the afternoon she had a chill and felt considerably exhausted,—as she expressed it, "I began to realize what I had been through." On the second day she felt somewhat better, but had another slight chill. On the third day there was a normal condition,—good appetite, patient was up, and the pain in the head had subsided considerably. For a week or ten days after the operation there was considerable discharge from the cavity, and the parts were very sensitive to the slightest cold. The treatment of the wound, from August 6 to 22, was done by the patient, Dr. Hitchcock seeing the case several times during that interval. After the eighteenth day the packing of the cavity was from day to day lessened, the wound was allowed to heal and fill up from the bottom, and on September 20 it was considered well.

